# Genome-Wide Association Study of Multiple Sclerosis Confirms a Novel Locus at 5p13.1

**DOI:** 10.1371/journal.pone.0036140

**Published:** 2012-05-03

**Authors:** Fuencisla Matesanz, Antonio González-Pérez, Miguel Lucas, Serena Sanna, Javier Gayán, Elena Urcelay, Ilenia Zara, Maristella Pitzalis, María L. Cavanillas, Rafael Arroyo, Magdalena Zoledziewska, Marisa Marrosu, Oscar Fernández, Laura Leyva, Antonio Alcina, Maria Fedetz, Concha Moreno-Rey, Juan Velasco, Luis M. Real, Juan Luis Ruiz-Peña, Francesco Cucca, Agustín Ruiz, Guillermo Izquierdo

**Affiliations:** 1 Unidad de Esclerosis Múltiple, Hospital Virgen Macarena, Sevilla, Spain; 2 Department of Structural Genomics, Neocodex, Sevilla, Spain; 3 Servicio de Biología Molecular, Hospital Virgen Macarena, Sevilla, Spain; 4 Istituto di Ricerca Genetica e Biomedica, Consiglio Nazionale delle Ricerche, Monserrato, Italy; 5 Immunology Department, H. Clínico S. Carlos, Instituto de Investigación Sanitaria S. Carlos (IdISSC), Madrid, Spain; 6 Center for Advanced Studies, Research and Development in Sardinia (CRS4), AGCT, Parco tecnologico della Sardegna, Pula, Italy; 7 Dipartimento di Scienze Biomediche, Università di Sassari, Sassari, Italy; 8 Multiple Sclerosis Unit, Neurology Department, H. Clínico S. Carlos, Instituto de Investigación Sanitaria S. Carlos (IdISSC), Madrid, Spain; 9 Dipartimento di Scienze Neurologiche e Cardiovascolari, Centro Sclerosi Multipla, Università di Cagliari, Cagliari, Italy; 10 Servicio de Neurología, Instituto de Neurociencias Clínicas, Hospital Regional Universitario Carlos Haya, Málaga, Spain; 11 Instituto de Parasitología y Biomedicina “López Neyra”, CSIC, Granada, Spain; Institute Biomedical Research August Pi Sunyer (IDIBAPS) - Hospital Clinic of Barcelona, Spain

## Abstract

Multiple Sclerosis (MS) is the most common progressive and disabling neurological condition affecting young adults in the world today. From a genetic point of view, MS is a complex disorder resulting from the combination of genetic and non-genetic factors. We aimed to identify previously unidentified loci conducting a new GWAS of Multiple Sclerosis (MS) in a sample of 296 MS cases and 801 controls from the Spanish population. Meta-analysis of our data in combination with previous GWAS was done. A total of 17 GWAS-significant SNPs, corresponding to three different loci were identified:HLA, IL2RA, and 5p13.1. All three have been previously reported as GWAS-significant. We confirmed our observation in 5p13.1 for rs9292777 using two additional independent Spanish samples to make a total of 4912 MS cases and 7498 controls (ORpooled = 0.84; 95%CI: 0.80–0.89; p = 1.36×10-9). This SNP differs from the one reported within this locus in a recent GWAS. Although it is unclear whether both signals are tapping the same genetic association, it seems clear that this locus plays an important role in the pathogenesis of MS.

## Introduction

Multiple Sclerosis (MS) is the most common progressive and disabling neurological condition affecting young adults in the world today. The overall prevalence of MS is about 70 per 100,000 individuals (range from 2 to 150) [1]. Pathogenetically, MS is considered an inflammatory and autoimmune disease with secondary demyelination during the first years of disease progress, which often presents clinically with relapsing/remitting symptoms. After this phase, axonal loss occurs contributing to a secondary progressive course of the disease [2].

From a genetic point of view, MS is a complex disorder resulting from the combination of genetic and non-genetic factors [3]. Human leukocyte antigen (HLA) at 6p21 has long been recognized as the strongest locus increasing risk to MS in most populations [4]. More recently, at least two independent signals within the HLA chromosomal region have been reported [5]. Other genetic factors involved in MS have remained elusive until the arrival of Genome-Wide Association studies (GWAS) which facilitated the identification of new susceptibility loci (The MSGene Database. http://www.msgene.org/).

We aimed to identify previously unidentified loci conducting a new GWAS of Multiple Sclerosis (MS) in a sample of 296 MS cases and 801 controls from the Spanish population. Meta-analysis of our data in combination with previous GWAS was also planned. (Figure S1)

## Results

We analysed this new Spanish GWAS (named as the Macarena dataset) independently, but also in combination with the IMSGC/WTCCC and the Gene MSA datasets, looking for consistent genetic factors associated in all datasets. Briefly, we genotyped 296 MS patients and 801 controls from Spain using the Affymetrix 250K Nsp I array as previously described [6]. After an extensive quality control, we performed principal components analysis (PCA) [7] and selected for analyses 286 MS cases and 767 controls described below. We also analyzed a dataset (named hereafter IMSGC/WT) based on 931 MS cases from the International Multiple Sclerosis Genetic Consortium (IMSGC) [8] and 2938 controls from the Wellcome Trust Case Control Consortium (WTCCC), who had been genotyped using Affymetrix 500K array [9]. After extensive quality control and PCA pruning, similar to what we applied to the Macarena dataset, a total 886 cases and 2933 controls were considered for downstream analyses. Furthermore, the Gene MSA study [10] was incorporated as a third study group. Given that the Gene MSA series had been genotyped using the Sentrix HumanHap550 BeadChip platform from Illumina, we imputed ∼2.5 million markers from HapMap2 to this dataset using PLINK [11]. Genotypes on associated SNPs were also corroborated with a second imputation methodology (Mach v 1.0) [12]. Effective sample size after PCA and genotype quality control for Gene MSA was 955 MS cases and 858 controls.

**Figure 1:**
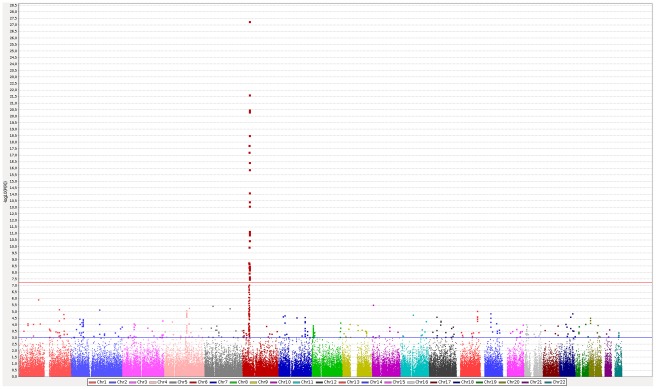
Manhattan plot of Meta-Analysis of three GWAS datasets (Macarena, IMSGC/WT, GeneMSA). Blue and red horizontal lines correspond to p values of 0.001 and 3.82×10-7 respectively.

**Figure 2:**
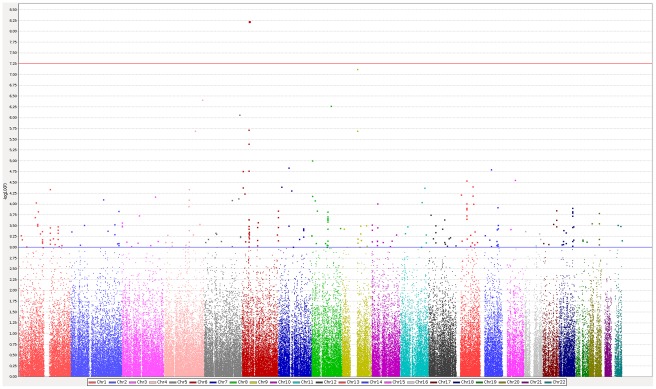
Manhattan plot of Macarena GWAS. Blue and red horizontal lines correspond to p values of 0.001 and 3.82×10-7 respectively.

We then performed a meta-analysis of these three GWAS datasets on the 130,903 shared SNPs (Figure 1 and Figure 2), and selected markers associated with a p-value below 0.005 in the random effects model for further analyses. While this threshold is much less stringent than the common GWAS threshold or that of a Bonferroni correction, we thought to promote a wider list of SNPs as in a two-stage analysis given the availability of an “in-silico” replication. A total of 1,064 SNPs meeting this criterion were indeed analyzed “in silico” in a recent GWAS performed on 882 MS cases and 872 controls from the Sardinian population [13]. After integrating these data in a new meta-analysis, we found that 17 markers reached the bonferroni significance of 3.82×10-7 (Table 1). Most of these markers (15 of them) were located within the *HLA* region, and the remaining two markers were located within the *IL2RA* gene (rs12722489, p = 2.16×10-7) and the 5p13.1 regulatory region near *PTGER4* (rs9292777, p = 9.84×10-9), respectively. Both *HLA* and *IL2RA* are well established MS risk loci (www.msgene.org). *PTGER4* has been previously found to be associated with Crohn's disease [9], [14], [15], [16], reported as “suggestive signal” in a meta-analysis of MS GWAS [17], and also reported as genome-wide significant for MS in a recent GWAS by the International Multiple Sclerosis Genetics Consortium/Wellcome Trust Case Control Consortium. The marker reported in this study (rs4613763) [18] is about 45 kb from our marker, with a D′ of 1 and r^2^ of 0.13, so they may not be necessarily tapping the same genetic association. Indeed a conditional analysis shows independent effects of rs9292777 in two of the three GWAS datasets (rs4613763 could not be imputed in Macarena dataset). Thus, after conditioning on rs4613763 the p-value of rs9292777 went from the original 0.0003 to 0.0039 in IMSGC/WTCCC and from 0.0019 to 0.0012 in Gene MSA. The corresponding p-values of rs4613763 before and after conditioning on rs9292777 in the same two GWAS went from 0.00186 to 0.064 and from 0.826 to 0.335 respectively.

**Table 1:** Ranking of genome-wide significant markers in the Meta-Analysis of four independent GWAS datasets (Macarena, IMSGC/WT, GeneMSA, and 1064 SNPs of Sardinian study).

						GWAS_Macarena	IMSGC/WT	GeneMSA	Sardinia	Meta-Analysis*	
LOCUS	CHR	BP	SNP	A1	A2	OR (95%CI)	OR (95%CI)	OR (95%CI)	OR (95%CI)	OR (95%CI)	P
HLA	6	32421075	rs4959093	C	T	0.61 (0.49–0.75)	0.65 (0.58–0.73)	0.62 (0.55–0.71)	0.70 (0.61–0.80)	0.65 (0.61–0.70)	8.18E-34
HLA	6	32776314	rs2647046	A	C	1.34 (1.10–1.63)	1.79 (1.60–1.99)	1.92 (1.68–2.19)	1.79 (1.54–2.08)	1.72 (1.52–1.95)	1.43E-17
HLA	6	31355046	rs7382297	T	G	1.46 (1.08–1.96)	1.86 (1.63–2.11)	1.65 (1.37–1.98)	1.34 (0.90–2.00)	1.68 (1.47–1.91)	2.01E-15
HLA	6	32174155	rs17421624	C	T	0.66 (0.53–0.84)	0.69 (0.60–0.78)	0.67 (0.58–0.77)	0.81 (0.69–0.94)	0.71 (0.65–0.78)	9.72E-15
HLA	6	30230554	rs2517646	C	T	1.08 (0.89–1.32)	1.29 (1.15–1.44)	1.30 (1.14–1.49)	1.34 (1.18–1.53)	1.28 (1.19–1.37)	1.95E-11
HLA	6	30217840	rs9261491	C	A	0.82 (0.63–1.06)	0.79 (0.70–0.90)	0.68 (0.58–0.80)	0.78 (0.65–0.93)	0.76 (0.70–0.83)	1.44E-10
HLA	6	30214275	rs2857439	A	G	0.73 (0.55–0.97)	0.79 (0.69–0.91)	0.67 (0.57–0.79)	0.78 (0.62–0.99)	0.74 (0.68–0.81)	1.86E-10
HLA	6	30134125	rs16896944	C	T	1.26 (0.89–1.78)	1.44 (1.07–1.94)	1.30 (0.82–2.05)	1.59 (1.34–1.88)	1.49 (1.31–1.69)	2.64E-09
HLA	6	30286266	rs3132671	T	C	0.79 (0.64–0.96)	0.78 (0.70–0.87)	0.81 (0.71–0.92)	0.91 (0.78–1.06)	0.81 (0.76–0.87)	3.78E-09
HLA	6	30214003	rs2857435	T	A	0.76 (0.58–1.01)	0.81 (0.71–0.93)	0.67 (0.57–0.79)	0.77 (0.61–0.97)	0.75 (0.69–0.83)	8.37E-09
PTGER4	5	40473705	rs9292777	C	T	0.92 (0.76–1.13)	0.82 (0.73–0.91)	0.81 (0.70–0.92)	0.79 (0.69–0.90)	0.82 (0.77–0.87)	9.84E-09
HLA	6	30213328	rs9261471	C	T	0.77 (0.58–1.02)	0.81 (0.71–0.92)	0.67 (0.57–0.79)	0.79 (0.62–1.00)	0.76 (0.69–0.83)	1.36E-08
HLA	6	31373518	rs3905495	A	G	0.73 (0.60–0.90)	0.86 (0.77–0.97)	0.78 (0.68–0.89)	0.84 (0.72–0.98)	0.81 (0.76–0.88)	4.76E-08
HLA	6	29812062	rs1736916	C	T	1.15 (0.89–1.49)	1.37 (1.20–1.55)	1.24 (1.05–1.47)	1.13 (0.87–1.47)	1.28 (1.17–1.40)	1.22E-07
HLA	6	29944218	rs3094157	C	G	1.21 (0.89–1.64)	1.38 (1.20–1.59)	1.24 (1.03–1.49)	1.25 (0.91–1.73)	1.31 (1.18–1.44)	1.24E-07
HLA	6	30222934	rs757262	T	C	0.78 (0.59–1.03)	0.82 (0.72–0.94)	0.67 (0.57–0.79)	0.78 (0.62–0.99)	0.76 (0.69–0.84)	1.75E-07
IL2RA	10	6142018	rs12722489	T	C	0.68 (0.49–0.95)	0.73 (0.62–0.85)	0.85 (0.70–1.03)	0.76 (0.60–0.97)	0.76 (0.69–0.84)	2.16E-07

* Summary estimate and p-value from Meta-analysis using random effects model including estimates from the four GWAS.

To further validate this marker we considered two independent samples from the Spanish population, namely the HCSC and the IPBLN samples. They included 646 and 1257 MS patients, and 746 and 1322 controls respectively.. These healthy controls were matched to cases by age, gender, ethnicity and place of recruitment [19]. The selected SNP rs9292777 was imputed in the HCSC sample and genotyped in the IPBLN sample, using Taqman technology as described elsewhere. The respective ORs for rs9292777 were 0.92 (95% CI: 0.79–1.07, p = 0.29) and 0.87 (95% CI: 0.78–0.98, p = 0.02).

**Figure 3:**
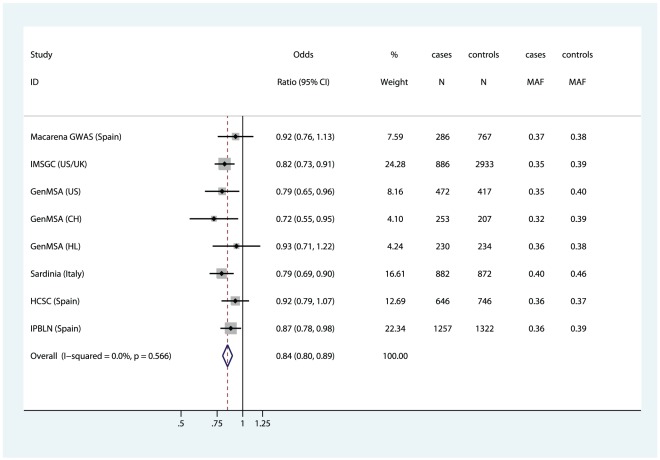
Forest plot of Meta-analysis including the GWAS datasets (with GeneMSA included as three independent substudies), and the validation samples. MAF: Minor Allele Frequency; US: United States; UK: United Kingdom; CH: Confoederatio Helvetica/Switzerland; HL: Holland.

All available evidence from the GWAS datasets and the validation samples were combined in a final meta-analysis that included a total of 4912 MS cases and 7498 controls. Overall, the C allele of rs9292777 was associated with an OR of 0.84 (95%CI: 0.80–0.89) corresponding to a p-value of 1.36×10-9, below the 5×10-8 Genome-wide significant cutoff. (Figure 3)

**Figure 4:**
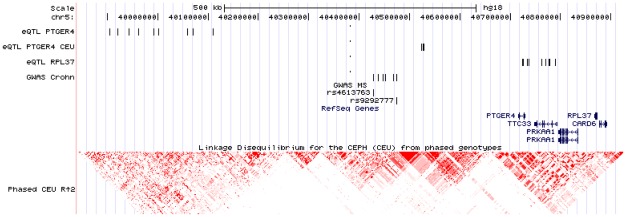
Location of eQTLs of the 5p13.1 region and the SNPs associated to MS and Crohn diseases. Image from the UCSC browser showing the chr5:39,840,547-40,924,217 (NCBI36/hg18) region. Vertical bars indicate the location of eQTLs and SNPs associated to Crohn or MS in different studies. eQTL PTGER4 are described by Zeller et al.; eQTL PTGER4 CEU are obtained from the correlation of PTGER4 expression in the CEU lymphoblastoid cell lines with the variants of the region; eQTL RPL37 are described by Stranger et al. and Dixon et al.; GWAS Crohn indicate the SNPs associated with the disease in different studies [9], [14], [15], [16]; GWAS MS indicate the SNPs associated with MS. LD plot performed with HapMap data from CEU population.

To further determine if the MS associated SNPs at the 5p13.1 locus could have a functional effect on the transcription of the genes located in the region, we assessed eQTLs in the chr5: 39885067-40883829 (NCBI37/hg19) locus by spearman rank correlation test for association between SNP genotypes of the region and mRNA levels of 5 genes in the region (*TTC33*, *PRKAA1*, *PTGER4*, *CARD6*, *RPL37*). The expression of the genes was assayed by Illumina Human-6 v2 Expression BeadChip with RNA samples from 109 lymphoblastoid cell lines from Utah residents with Northern and Western European ancestry from the CEPH collection (HapMap CEU population). Since the CEU samples were sequenced by the 1000 Genomes project, we downloaded the variant information from the 1000 Genomes database. 2593 SNPs and 671 insertion/deletion variants with a MAF>0.05 were correlated with the expression of the 5 genes. Significant thresholds for each population were assigned through 1000 permutations. At the 0.05 permutation threshold, we observed 2 SNPs that were significantly associated with *PTGER4* expression (rs7725052 and rs7714574) (Table S1). These 2 SNPs are in almost total linkage disequilibrium (LD)and are located 193-187 Kbp from the *PTGR4* gene. Other eQTLs in the region reported by other authors were also explored. Several SNPs located in position chr5:39,904,511-40,111,728 (NCBI37/hg19) have been reported to be eQTLs for *PTGER4* by Zeller et al [20]. These eQTLs are within 605 to 811 Kbp from the *PTGR4* gene and are different SNPs than the eQTLs found by us in the CEU samples. Expression QTLs for the *RPL37* gene in the region chr5:40,723,806-40,790,598 (NCBI37/hg19) were also reported by Stranger et al. [21] and by Dixon et al. [22] as shown in Figure 4. Since none of the MS associated SNPs were eQTLs for any of the genes in the region (Figure S2), we analyzed their LD with the associated variants. There is no LD between the distal *PTGER4* eQTLs, the *RPL37* eQTLs and the SNPs reported associated with Crohn by GWAS or the variants associated with MS (Figure S3). However, the *PTGER4* eQTLs obtained in the CEU population were in LD with the rs9292777 (r^2^ = 0.23). A lower LD was observed for the rs4613763 (r^2^ = 0.14) variant. None of the *PTGER4* proximal eQTLs were genotyped in any of the GWAS analyzed in the present work. We could impute data for rs7725052 in the Macarena and GeneMSA datasets. The ORs were 0.8403 and 0.8968 respectively.

## Discussion

We have analysed a total 130,903 markers using data from three GWAS and conducted several replications to detect a new marker associated with MS. While the failure to use imputation methods to increase the number of markers to be assessed might have restricted the power of our study (i.e. novel signals of markers that were not genotyped in the original GWAS would be missed), by no means it invalidates our findings. Our main finding, rs9292777 SNP, is located within an intergenic region on 5p13.1 [23]. As mentioned, this chromosomal region has been unequivocally associated (i.e. meta-GWAS significance, p<5e-8) with two autoimmune bowel diseases i.e. Crohn's disease and ulcerative colitis [9], [14], [15], [16]. Furthermore, a recent meta-GWAS presented preliminary evidence that this 5p13.1 locus could be associated to MS [17]. Specifically, the authors observed a signal for MS located at rs6896969 (p = 2×10-7). According to the 1000 genomes project data, and employing the SNAP software, rs6896969 and rs9292777 are 13522 bp away and can be considered reciprocal proxies (r^2^ = 0.901, D′ = 1) [24]. Therefore our results extend previous observations and confirm unequivocally the protective role of rs9292777, located on the 5p13.1 gene desert, for MS phenotype.

eQTL analysis of the 5p13.1 region revealed that some SNPs in LD with the MS associated variant rs9292777 correlated with the expression levels of the *PTGER4* gene. This expression association has been previously suggested by Lebouille et al. who observed that polymorphisms within this gene desert could modulate the expression levels of *PTGER4* gene, located 270 kb centromeric to rs9292777 [14]. *PTGER4* encodes the prostaglandin receptor EP4 which is a strong candidate susceptibility gene for inflammatory bowel diseases. In fact, *ptger4* knock-out mice develop severe colitis upon dextran sodium sulphate treatment [14]. Most importantly, recent experiments suggested that *ptger4* knock-out mice are protected against experimental autoimmune encephalomyelitis (EAE) signs compared with control mice [25]. Notably, EAE has proven to be a valuable model in elucidating pathogenesis as well as identifying candidate therapies for MS [25]. So, combining genetic and molecular data, PTGER4 pharmacological modulation appears as an excellent strategy for new drug development in human autoimmune diseases including MS.

## Materials and Methods

### Samples

In the present study we have analyzed three independent genomewide datasets: Macarena, IMSGC/WT and GeneMSA datasets and three validation collections: Sardinian, HCSC and IPBLN samples.

The Macarena dataset consisted of a genomewide genotyping effort (Affymetrix GeneChip Human Mapping 250K Nsp Array) including 293 definite MS cases and 801 controls, all natives of Spain [6]. MS cases were recruited during routine hospital visits to the MS unit at Virgen Macarena Universitary Hospital and diagnosed according to Poser and MacDonald criteria [26], [27]. Oligoclonal IgG intrathecal secrection in CSF and/or at least 3 out of 4 MRI Barkhof criteria was also considered necessary for inclusion in the study. All participants gave written informed consent under Macarena Hospital Institutional Review Board. DNA extraction from blood, PCR amplification, and genotyping was performed at Neocodex (Seville, Spain).

The second dataset (IMSGC/WT) consisted of MS cases from the International Multiple Sclerosis Genetics Consortium (IMSGC) and controls from the Wellcome Trust case-control Consortium (WTCCC). We employed the NINDS dbGaP dataset that includes genomewide genotypes (Affymetrix GeneChip Human Mapping 500K Array Set) for 931 individuals with MS from the IMSGC [8], all drawn from the UK and the USA. These MS cases were matched to a control dataset (n = 2938) from the WTCCC, deposited in the European Genotype Archive (EGA) and genotyped with the same chip [9]. All WTCCC controls are >99% of European origin living in the UK.

We have also employed the dbGaP GeneMSA dataset, consisting of genomewide genotypes (Illumina Sentrix HumanHap 550K BeadChip) for 980 MS cases and 900 controls [10], [28], drawn from the USA, the Netherlands, and Switzerland.

Although these downloaded datasets had already undergone quality control (QC) and stratification filters by the original authors, we performed an extra layer of QC analyses on them (described below).

In order to validate the most significant results (top 1064 SNPs with p-value<0,005) of a meta-analysis of the 3 GWAS datasets, we obtained aggregated frequency data and performed a first *in-silico* replication in an independent dataset of 882 MS patients and 872 controls from Sardinia, Italy [13].

The top novel SNP associated with MS was genotyped or imputed in two independent validation samples from Spain. The first sample (HCSC) includes 557 Spanish MS patients and 799 ethnically matched controls, mostly blood donors and staff, who were consecutively recruited from the Hospital Clínico San Carlos (Madrid). Patients were diagnosed based on the Poser criteria and included in the study after informed consent. Clinical features in the MS cohort from Madrid (35% men and 65% women): mean age at onset of 29 years and age of the patients ranging from 16 to 79 years, 36% of them were carriers of the major susceptibility factor for MS (HLA-DRB1*1501). The mean Expanded Disability Status Scale (EDSS) was 3.5 and approximately 10% of the patients had father, mother or a sibling with MS. The clinical forms distributed as follows: 7% Primary Progressive, 10% Secondary Progressive and 75% Relapsing-Remitting. The Ethics Committee of this Hospital approved the study.

The second sample (Instituto de Parasitología y Biomedicina Lopez-Neyra, IPBLN) comprises 1282 MS cases and 1322 controls from 4 hospitals and a blood bank of Andalucía, specifically, Hospital Virgen Macarena of Sevilla, Hospital Carlos Haya of Málaga, Hospital Clínico Virgen de las Nieves of Granada and the Blood Bank of Granada. The mean age at sample collection of the cases was 29.84 (10.66 sd) years and mean age of controls at interview was 33.43 (12.19 sd) years. The percentage of females was 68% for cases and 68% for controls. All MS cases were classified as RR (relapsing-remitting) or SP (secondary progressive). All patients were ascertained to have definite MS according to the Poser or MacDonald criteria [26], [27]. The study was approved by the Ethics Committees of each of the hospitals participating in the study and written informed consent was obtained from all participants. Genotyping was performed using the Taqman® SNP Genotyping Assay (Applied Biosystems) C_30119807_10 (rs9292777)

#### Quality Control (QC) analyses

We performed an extensive quality control on these datasets, using Affymetrix Genotyping Console software (http://www.affymetrix.com) and Plink [11]. Only individuals with a sample call rate above 93% were later re-called with the Bayesian Robust Linear Model with Malalanobis (BRLMM) distance algorithm, ran with default parameters, which improves call rates in most samples. Self-reported sex was compared to sex assigned by chromosome X genotypes, and discrepancies were resolved or samples removed. The program Graphical Representation of Relationships (GRR) [29] was used to check sample relatedness and to correct potential sample mixups, duplications, or contaminations. SNPs were selected to have a call rate above 95% (in each case, control, and combined group, within each dataset), and a minor allele frequency above 1% (again in each case, control, and combined group, within each dataset). SNPs that deviated grossly from Hardy-Weinberg equilibrium (p-value<10^−4^) in control samples were also removed. We also removed SNPs with a significantly different rate of missingness (p-value<5×10^−4^) between case and control samples within each dataset.

To ensure all SNPs across all datasets were typed according to the same DNA strand, each dataset was normalized using HapMap as the reference set. We merged each study with the HapMap CEU samples and compared genotype calls. SNP calls were flipped (if typed on opposite strand) or removed (if strand could not be undoubtedly assigned) as necessary. We also removed SNPs that were significantly associated with “study status”. That is, we labeled individuals from each study as cases and HapMap CEU individuals as controls, and removed SNPs with p-values smaller than 10-6 in a test for association.

#### Principal Components (PC) analysis

Principal components analysis was carried out with EIGENSOFT [7], [30], to evaluate population admixture within each population, and to identify individuals as outliers. We ran the SMARTPCA program with default parameters, excluding chromosome X markers. To minimize the effect of LD in the analysis, we also excluded markers in high LD (with the indep-pairwise option in Plink) and long-range LD regions reported previously or detected in our population. Individuals identified as outliers (6 standard deviations or more along one of the top ten principal components) were removed from all subsequent analyses. PC analysis was run within each dataset, and also together with other HapMap European and worldwide populations to detect individuals of different ethnicities. Overall, we removed 7 cases and 34 controls from the Macarena study, 45 cases and 5 controls from the IMSGC/WTCCC dataset, and 25 cases and 42 controls from the GeneMSA project.

### Imputation

Because two of the three datasets applied Affymetrix genotyping technology, we imputed the Affymetrix SNPs on all the participants in the GeneMSA study. Using the original Illumina genotypes for the GeneMSA participants, and HapMap reference genotypes (60 unrelated CEU samples), we imputed all of the Affymetrix 500K SNPs using two different methodologies: Plink [11] and MaCH [31]. Genotype calls with high quality scores (info>0.8 in Plink and r^2^>0.3 in MaCH) and consistently called with both methodologies were used in subsequent association analyses.

After all these quality control and preparatory steps, the Macarena study kept 195,035 SNPs for 286 cases and 767 controls; the IMSGC/WTCCC dataset kept 312,869 SNPs for 886 cases and 2933 controls; and the GeneMSA dataset contained 2,649,462 SNPs in 955 cases and 858 controls. The three datasets had 130,903 SNPs in common, which were then used in association analyses. Additionally, rs9292777 was imputed in the HCSC sample from surrounding SNPs available on this sample using the same methodology described above.

#### Association Analysis

Single-locus allelic (1 df) association analysis within each independent GWAS sample, and of the combined sample, was carried out using Plink [11]. The genomic inflation factor was also estimated, with Plink, for each dataset (Macarena:1.13; GenMSA:1.02; IMSGC/WT:1.16). We also carried out a meta-analysis of all three datasets using Plink. The top 1064 most significant SNPs (p<0.005) resulting from this meta-analysis were selected for replication using aggregated frequency data from an Italian sample, and performed a meta-analysis of these SNPs in the four populations. The resulting novel SNP (rs9292777) was imputed and genotyped respectively in two independent samples ascertained from the Spanish population. A final meta-analysis and Forest plot for this marker including estimates from the GWAS datasets, plus the validation samples was done with Stata 10.0 (College Station, TX) metan command.

### Expression association analysis and multiple test correction

We used Spearman's correlation to test for associations in *cis* between SNP genotypes and probe expression levels in each population. This method has been previously shown to produce robust results and avoids the effect of outliers in gene expression values [32]. We analyzed the SNPs from the 1000 Genomes project included between chr5: 39885067- 40883829 (Build 37). Significance thresholds for each gene were assigned after 1000 permutations of expression values relative to genotypes. Computation of the Spearman correlation test has been performed with Genetranssoc (http://bios.ugr.es/Genetranassoc/), a c++ software with an analogous implementation of the Spearman coefficient in the statistical package R which also computes statistical significance using permutation tests.Reported eQTL were assessed by http://eqtl.uchicago.edu/cgi-bin/gbrowse/eqtl/.

## Supporting Information

Figure S1 **Samples used in the different analyses.** This flow-chart describes the samples used in the different analyses. It starts with three initial GWAS which after extensive quality control yielded a total of 2,127 cases and 4,558 controls. Meta-analysis using 130,903 SNPs common to these samples was done and SNPs with a p value below 0.001 in this analysis were requested from the Sardinian GWAS. A new meta-analysis was done adding these new data. Only previously unreported SNPs with a p-value below 3.82×10-7 in this analysis were chosen for a final validation. Only one marker (rs929777) met the criteria and was analysed in a final meta-analysis that included the previous four samples and two new validation samples (HSCS and IPBLN). The final result of this analysis is the main finding of our study (rs929777, OR_pooled_ = 0.84; 95%CI: 0.80–0.89; p = 1.36×10-9).
(PPTX)

Figure S2 Plots of the *PTGER4* expression levels respect to the genotypes of two MS-risk variants and the *PTGER4* eQTL. In all plots, expression levels are represented for the three genotype groups. Box plots of expression data from normalized results of ILMN_1795930 (*PTGER4*) probe generated by Illumina Human-6 v2 Expression BeadChip (EMBL-EBI database, http://www.ebi.ac.uk/arrayexpress/, ID projects E-MTAB-198). P-values are calculated by Kruskal Wallis Test.
(TIF)

Figure S3 LD plots of the **5p13.1 region** eQTLs **and the** GWAS- SNPs associated with Crohn and MS. Data are from HapMap III CEU population. LD by r^2^.
(TIF)

Table S1 Spearman correlation test of variants associated with *PETGR4* expression in different human populations.
(XLSX)
