# Supplementary material for: Genome-Wide Association Study of Multiple Sclerosis Confirms a Novel Locus at 5p13.1
Source: PLoS One. 2012 May 3;7(5):e36140. doi: 10.1371/journal.pone.0036140 (PMC3343041; doi:10.1371/journal.pone.0036140)
Supplement: Figure S1 — Samples used in the different analyses. This flow-chart describes the samples used in the different analyses. It starts with three initial GWAS which after extensive quality control yielded a total of 2,127 cases and 4,558 controls. Meta-analysis using 130,903 SNPs common to these samples was done and SNPs with a p value below 0.001 in this analysis were requested from the Sardinian GWAS. A new meta-analysis was done adding these new data. Only previously unreported SNPs with a p-value below 3.82×10^-7^ in this analysis were chosen for a final validation. Only one marker (rs929777) met the criteria and was analysed in a final meta-analysis that included the previous four samples and two new validation samples (HSCS and IPBLN). The final result of this analysis is the main finding of our study (rs929777, ORpooled = 0.84; 95%CI: 0.80–0.89; p = 1.36×10^-9^). (PPTX) [file pone.0036140.s001.pptx]

## Slide 1
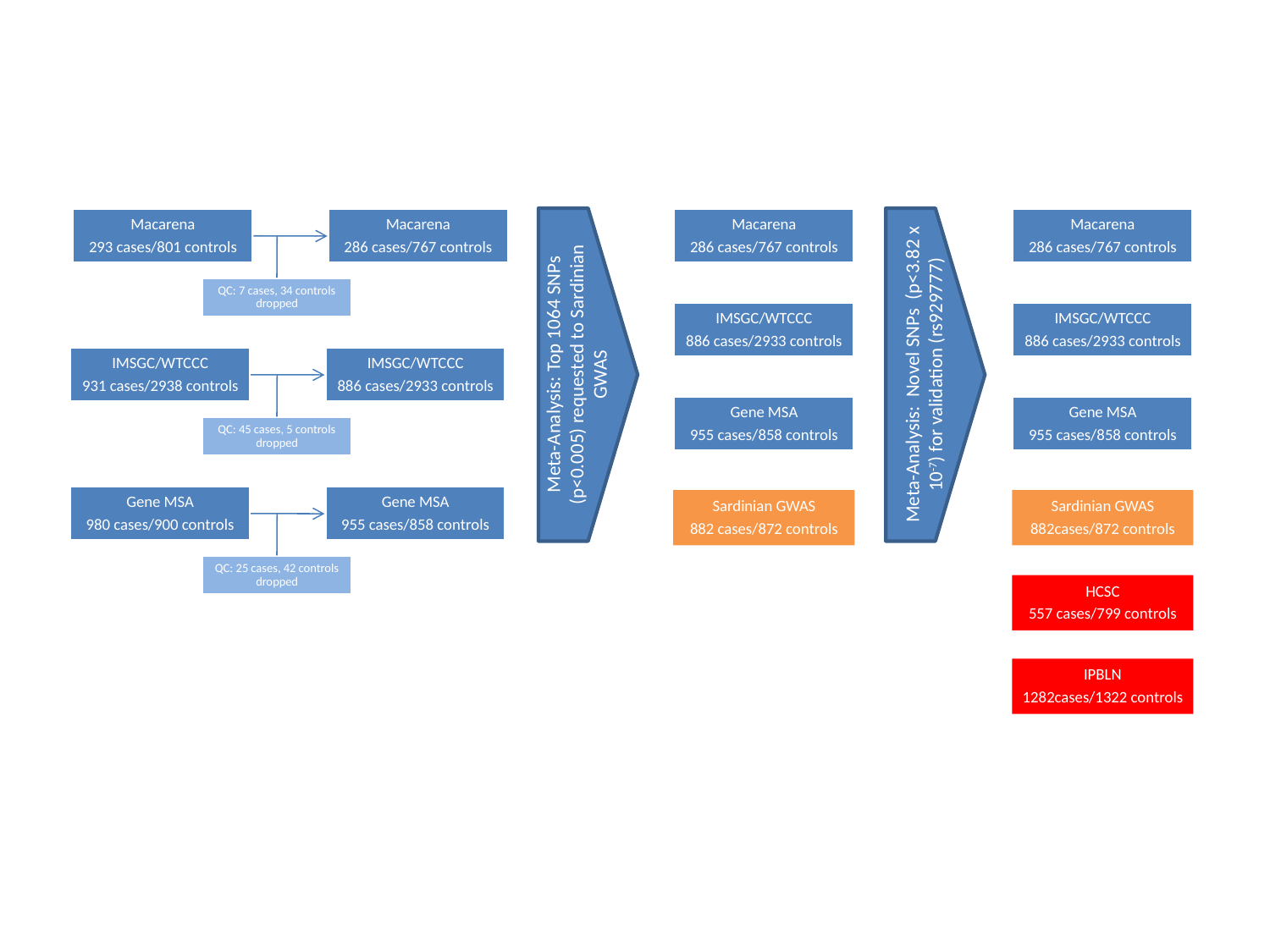

Macarena
293 cases/801 controls
Macarena
286 cases/767 controls
Meta-Analysis: Top 1064 SNPs (p<0.005) requested to Sardinian GWAS
Macarena
286 cases/767 controls
Meta-Analysis: Novel SNPs (p<3.82 x 10^-7^) for validation (rs929777)
Macarena
286 cases/767 controls
QC: 7 cases, 34 controls dropped
IMSGC/WTCCC
886 cases/2933 controls
IMSGC/WTCCC
886 cases/2933 controls
IMSGC/WTCCC
931 cases/2938 controls
IMSGC/WTCCC
886 cases/2933 controls
Gene MSA
955 cases/858 controls
Gene MSA
955 cases/858 controls
QC: 45 cases, 5 controls dropped
Gene MSA
980 cases/900 controls
Gene MSA
955 cases/858 controls
Sardinian GWAS
882 cases/872 controls
Sardinian GWAS
882cases/872 controls
QC: 25 cases, 42 controls dropped
HCSC
557 cases/799 controls
IPBLN
1282cases/1322 controls
