# Supplementary figures and images for: Genome-Wide Association Study of Multiple Sclerosis Confirms a Novel Locus at 5p13.1
Source: PLoS One. 2012 May 3;7(5):e36140. doi: 10.1371/journal.pone.0036140 (PMC3343041; doi:10.1371/journal.pone.0036140)

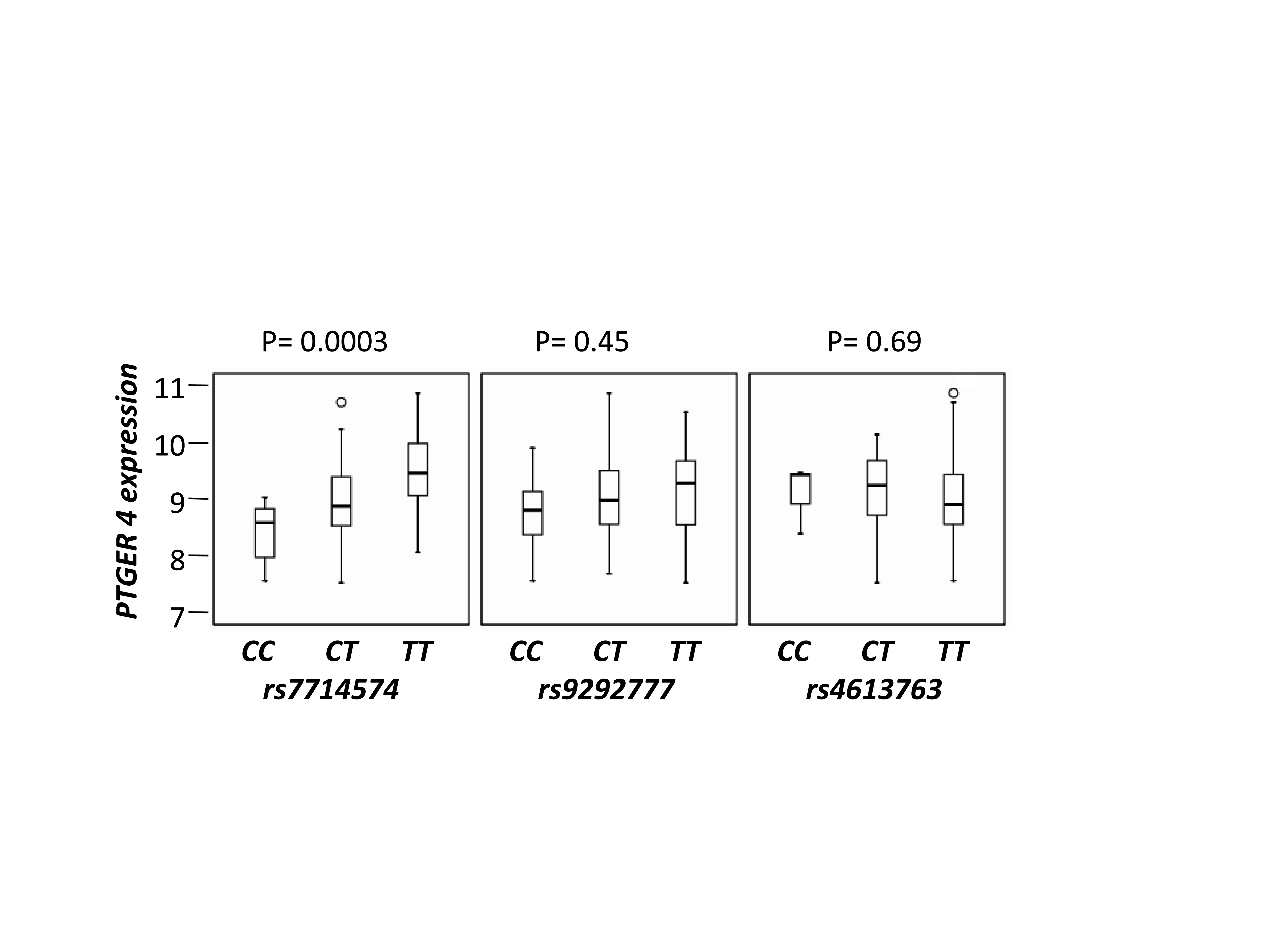

Supplement: Figure S2 — Plots of the PTGER4 expression levels respect to the genotypes of two MS-risk variants and the PTGER4 eQTL. In all plots, expression levels are represented for the three genotype groups. Box plots of expression data from normalized results of ILMN_1795930 (PTGER4) probe generated by Illumina Human-6 v2 Expression BeadChip (EMBL-EBI database, http://www.ebi.ac.uk/arrayexpress/, ID projects E-MTAB-198). P-values are calculated by Kruskal Wallis Test. (TIF) [file pone.0036140.s002.tif]

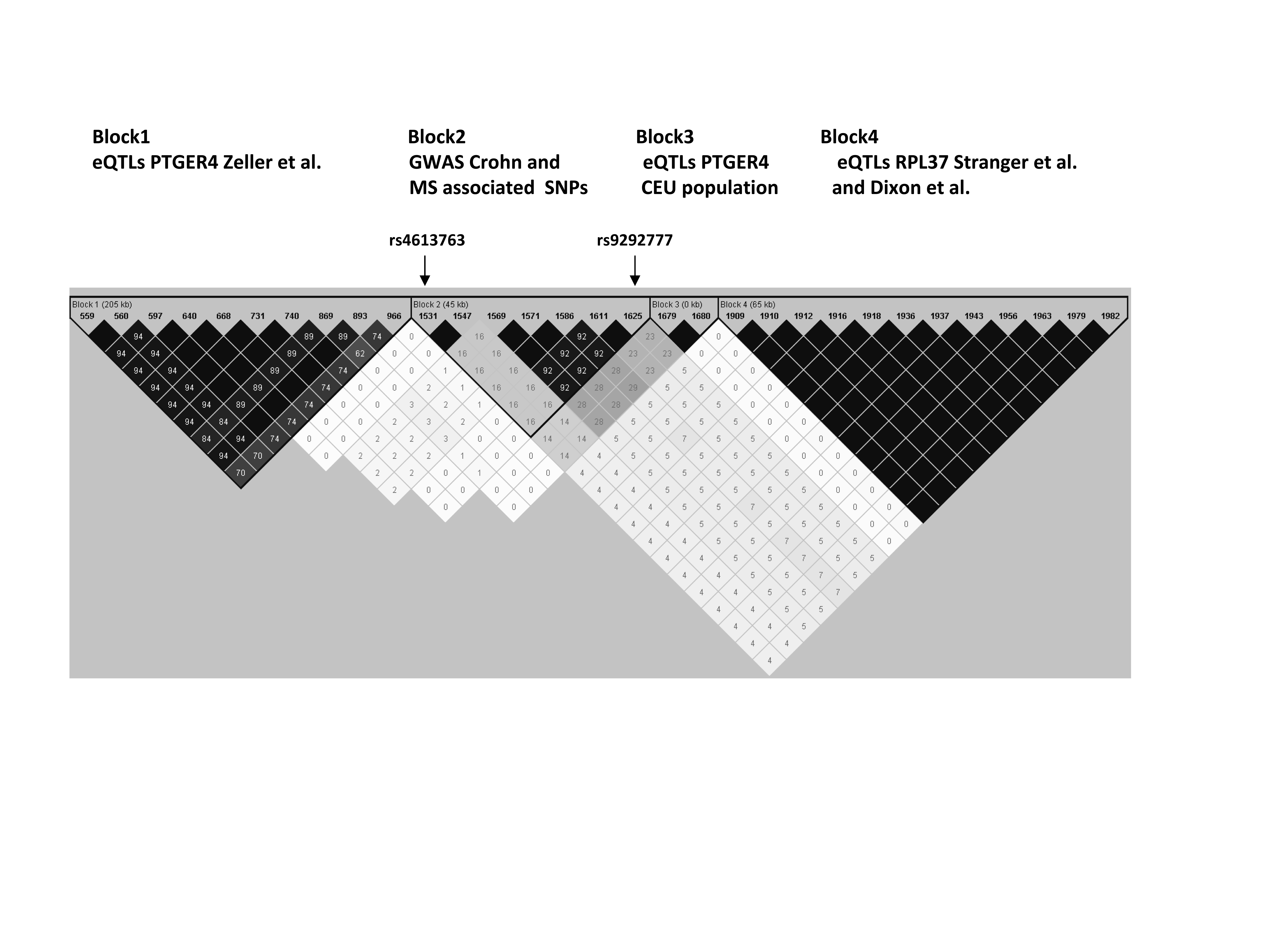

Supplement: Figure S3 — LD plots of the 5p13.1 region eQTLs and the GWAS- SNPs associated with Crohn and MS. Data are from HapMap III CEU population. LD by r2. (TIF) [file pone.0036140.s003.tif]
